# Coupling Machine Learning and Lipidomics as a Tool to Investigate Metabolic Dysfunction-Associated Fatty Liver Disease. A General Overview

**DOI:** 10.3390/biom11030473

**Published:** 2021-03-22

**Authors:** Helena Castañé, Gerard Baiges-Gaya, Anna Hernández-Aguilera, Elisabet Rodríguez-Tomàs, Salvador Fernández-Arroyo, Pol Herrero, Antoni Delpino-Rius, Nuria Canela, Javier A. Menendez, Jordi Camps, Jorge Joven

**Affiliations:** 1 Unitat de Recerca Biomèdica (URB-CRB), Hospital Universitari de Sant Joan, Institut d’Investigació Sanitària Pere Virgili, Universitat Rovira i Virgili, C. Sant Joan s/n, 43201 Reus, Spain; helena.castane@urv.cat (H.C.); gerard.baiges@iispv.cat (G.B.-G.); anna.hernandez@iispv.cat (A.H.-A.); elisabet.rodriguez@urv.cat (E.R.-T.); 2 Eurecat, Centre Tecnològic de Catalunya, Centre for Omic Sciences (Joint Unit Eurecat-Universitat Rovira i Virgili), Unique Scientific and Technical Infrastructure (ICTS), Av. Universitat 1, 43204 Reus, Spain; salvador.fernandez@eurecat.org (S.F.-A.); pol.herrero@eurecat.org (P.H.); antoni.delpino@eurecat.org (A.D.-R.); nuria.canela@eurecat.org (N.C.); 3 Girona Biomedical Research Institute (IDIBGI), C. Dr. Castany s/n, 17190 Salt, Spain; jmenendez@idibgi.org

**Keywords:** adipose tissue, artificial intelligence, bariatric surgery, deep learning, metabolism, NASH

## Abstract

Hepatic biopsy is the gold standard for staging nonalcoholic fatty liver disease (NAFLD). Unfortunately, accessing the liver is invasive, requires a multidisciplinary team and is too expensive to be conducted on large segments of the population. NAFLD starts quietly and can progress until liver damage is irreversible. Given this complex situation, the search for noninvasive alternatives is clinically important. A hallmark of NAFLD progression is the dysregulation in lipid metabolism. In this context, recent advances in the area of machine learning have increased the interest in evaluating whether multi-omics data analysis performed on peripheral blood can enhance human interpretation. In the present review, we show how the use of machine learning can identify sets of lipids as predictive biomarkers of NAFLD progression. This approach could potentially help clinicians to improve the diagnosis accuracy and predict the future risk of the disease. While NAFLD has no effective treatment yet, the key to slowing the progression of the disease may lie in predictive robust biomarkers. Hence, to detect this disease as soon as possible, the use of computational science can help us to make a more accurate and reliable diagnosis. We aimed to provide a general overview for all readers interested in implementing these methods.

## 1. Introduction

Deep knowledge of disease pathogenesis facilitates diagnosis even with a trivial amount of data and improves the design of clinical research to discover the appropriate cure. Underdiagnosis and undefined clinical predictions characterize nonalcoholic fatty liver disease (NAFLD), which are major factors influencing the lack of licensed drug treatment and efficacious management. Hypothesis-driven research has provided advances in knowledge, but the method is slow and severely constrained when diagnosis requires accurate histological diagnosis.

Major breakthroughs in medicine resulting from chance or serendipity are extremely unlikely, as illustrated by fascinating anecdotes (e.g., sildenafil, penicillin, antidepressants or the actual cause of most gastric ulcers), but today’s technology enables scientific research to advance through untargeted methods, exploring at random and generating enormous datasets. For example, and to honor the recent Nobel laureates in Chemistry, it is illustrative to consider the high throughput computational and experimental data leading to the discovery that up to 20% of all bacterial genes code for critical regulatory RNAs [1]. Subsequent hypothesis-driven research has driven the discovery of complex genomic architectures of the clustered regularly interspaced short palindromic repeats (CRISPR)-associated proteins (CRISPR-Cas) systems [2] and the specific function of Cas9 (an RNA guided-DNA endonuclease) [3]. The ultimate results are paving the way toward essential discoveries [4]. The ability to collect and analyze large datasets related to medical outcomes promises to transform medicine, providing a future of personalized treatments. Here, we summarize the challenges faced by lipidomics to provide knowledge, noninvasive diagnosis and predictions of stage in metabolic dysfunction-associated fatty liver disease (MAFLD), as well as the associated methods of machine learning analysis that are an absolute requirement to obtain robust results. 

## 2. NAFLD Is a Major Global Health Challenge: A Silent Pandemic

**Figure 1:**
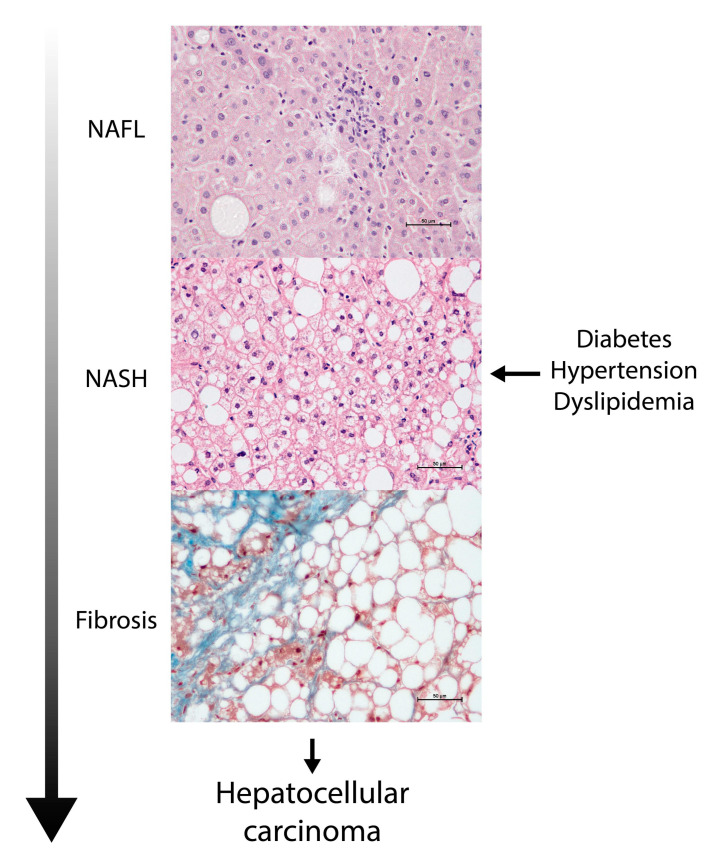
Nonalcoholic fatty liver disease is apparently progressive. The accumulation of fat in the liver may be considered clinically as a serious event, especially when accompanied or caused by metabolic dysregulation. Liver assessment is commonly underdiagnosed in the management of the metabolic syndrome with potential deleterious consequences.

Obesity is the most rapidly global health concern and is closely linked to cardiovascular and oncologic deaths through metabolic dysfunction, diabetes, dyslipidemia, hypertension and NAFLD. The prevalence estimates of NAFLD vary geographically, indicating the importance of genetic and environmental factors, but it apparently affects more than 20% of adults worldwide. No country is immune to the harms from NAFLD, and it is now the most prevalent liver disease in human history. Hepatic fat accumulation is the hallmark of NAFLD, and the disease may progress from simple steatosis or nonalcoholic fatty liver (NAFL) to necro-inflammatory forms, marked by hepatocyte ballooning, inflammation (nonalcoholic steatohepatitis; NASH) and fibrosis with cirrhosis; end-stage liver disease; or hepatocellular carcinoma as potential outcomes [5,6]. The actual mechanisms leading to progression are poorly understood, but it is more likely in the presence of metabolic dysfunction (Figure 1), which has led to a recent revision in the nomenclature (metabolic-associated fatty liver disease, MAFLD) and includes positive diagnostic criteria, rather than having a diagnosis of exclusion [7]. 

Not all patients experience morbidity, but prevalence is increasing, and predictions include NAFLD as a leading cause of liver transplantation, hepatocellular carcinoma and liver-related mortality [8]. The population at risk is extremely large and notably underdiagnosed, because the accurate diagnosis requires a liver biopsy, which is not completely safe and requires expensive resources. In contrast to other noncommunicable diseases (NCDs), NAFLD has been absent from public health strategies, including those focused on other NCDs that are intricately correlated with its causes and effects. This is probably because of the lack of histopathological examination results in biased knowledge of disease natural history and assessment of factors associated with progression [9]. Due to all these reasons and, considering that an early diagnosis of NAFLD and NASH can reverse the damage in the liver, the search for noninvasive biomarkers of disease severity, risk stratification, and monitoring of patients is an important goal [10]. As hepatic fat accumulation and the associated mitochondrial dysfunction, lipotoxicity and oxidative stress disrupt lipid metabolism, lipidomics might contribute to the foremost goal of integrating intra-individual heterogeneity with clinical uncertainties and to support the epidemiologic response to this silent health challenge [8,9,10].

## 3. Lipidomics, A Latecomer Omics Technology, Is Being Consolidated

Lipids are among the most important biomolecules, as major components of membranes with crucial tasks in energy distribution and the regulation of extra- and intracellular signaling processes [11]. Dysregulation of lipid composition in hepatocytes accumulate toxic lipids, and lipotoxicity contributes to the mitochondrial dysfunction, inflammation and deficient liver regeneration that are commonly found in NAFLD [5]. Lipids are, therefore, candidate molecules for intensive analysis in the disease, but complexity is a substantial challenge. As they have been ambiguously defined over the years, no exact definition of the term “lipid” exists. Some attempts have been made to classify them (i.e., LipidLibrary, Cyberlipid, LipidPedia, LipidBank), but the most accepted classification is currently provided by LIPID MAPS Structure database [11]. According to LIPID MAPS, eight different categories, with classes and subclasses, comprise more than 45,000 unique lipid structures. Advances in mass spectrometry allow high-throughput lipidomics methods, and analysis strategies necessitate an adequate coupling between computational data processing tools and the large volume of biological information. According to Pubmed, the number of articles published on lipidomics is relatively low, as compared with other omics technologies, but listed publications have grown exponentially in the last two years. Similarly, the number of listed publications on lipidomics and NAFLD in 2019 and 2020 is higher than those published since the pioneering work of Puri et al. [12] in 2007. Only a few studies, with not completely consistent results, have been conducted in humans, because the clinical difficulties in research persist, and the absolute need of liver biopsy remains a formidable limiting factor [13]. It is an appealing assumption that lipids in blood may propitiate the noninvasive assessment, liquid biopsy testing, of fat accumulation in the liver of NAFLD patients. Most challenges inherent to the analytical, technology-driven discipline of lipidomics may be circumvented, but difficulties in research and the tools for proper management must be identified.

## 4. Lipids Form a Heterogeneous and Complex Group of Small Molecules

**Figure 2:**
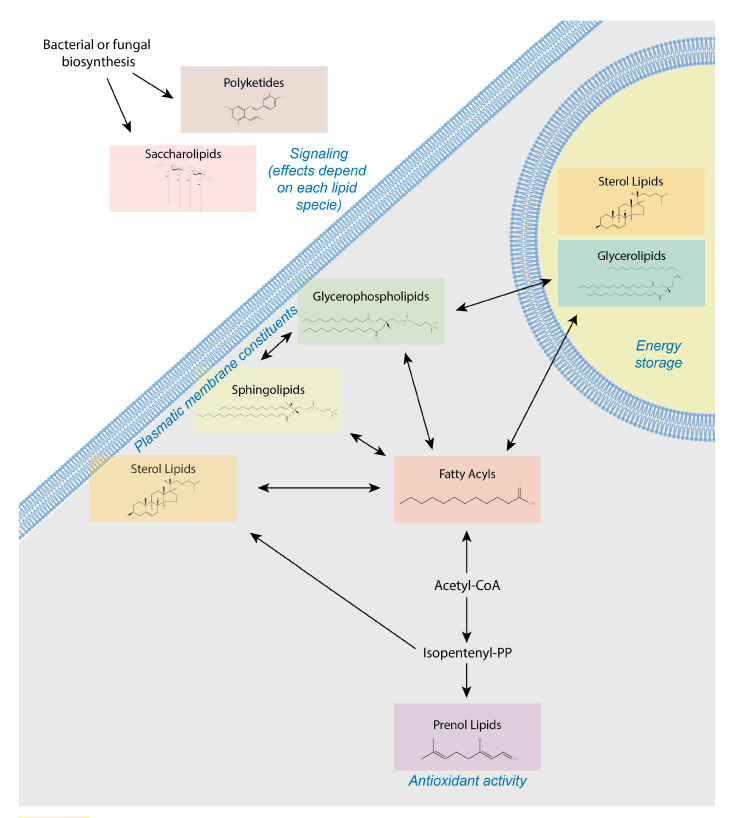
Lipotoxicity is an essential factor in the pathogenesis of liver disease. Actual mechanisms remain unknown, but the functional and structural organizations in membranes depend not only on the amount of accumulated fat but on the relative contribution of altered lipid composition and metabolism.

It is necessary to link changes in the blood to liver health, but the analysis requires careful evaluation of patterns and may be masked by variations in the health of other organs and associations with age, sex and environmental exposures. The first consideration is the complex structural organization of lipids. Specific lipid classes, and not the total amount of lipids stored in hepatocytes, may be the actual determinants of lipotoxicity. Succinctly, LIPID MAPS define eight different categories that can be chemically generated by either carbanion-based condensation of ketoacyl thioesters (fatty acyl, glycerolipids, glycerophospholipids, sphingolipids, saccharolipids and polyketides) or by carbocation-based condensation of isoprene units (prenol lipids and sterol lipids) (Figure 2). 

**Figure 3:**
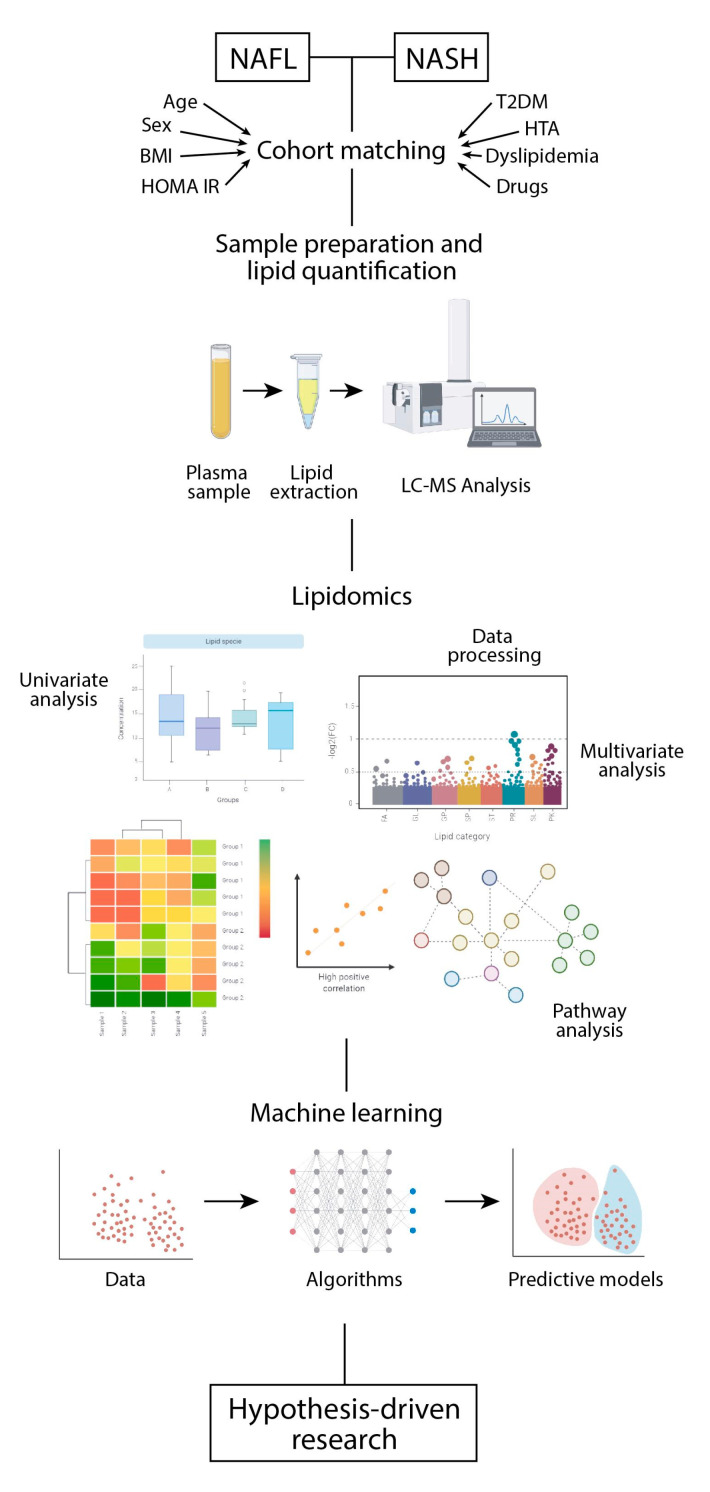
Lipidomics may propel the noninvasive assessment of progressive liver disease. Schematics illustrating important factors in the design of case-control studies aimed to understand the pathogenesis of nonalcoholic steatohepatitis (NASH). As part of the metabolic syndrome, cohort matching is an essential task that considers potential covariates. The management of samples and analytical procedures are crucial to provide high quality data. Lipidomics analysis results in an enormous amount of data that require the use of computers in each step of analysis and machine learning methods may ultimately result in predictive models. Created with BioRender.com (BioRender, Toronto, ON, Canada) https://biorender.com. Accessed 11 August 2020.

Fatty acyls are normally used as building blocks and represent repetitions of methylene groups in chains of four to twenty-four carbons with different number of double bounds and functionality [14]. Glycerolipids contain glycerol and triglycerides and exemplify energetic reservoirs, but diglycerides may be also active in biochemical signaling as second messengers and protein kinase C activators [15]. Glycerophospholipids, or simply phospholipids, and their derivatives, lysophospholipids, are classified according to the polar head, mainly choline, inositol, ethanolamine and serine and the characteristics of fatty acids attached to glycerol. Phospholipids are essential components of cell membranes but in blood may bear regulatory functions [16]. The physiological roles of sphingolipids are currently emerging [17] but are essential components in membranes. With a sphingoid base in their structure, they may be separated into sphingosines, phytosphingosines, ceramides, sphingomyelins, acylceramides and phosphonosphingolipids. In contrast, the functions of sterol lipids (cholesterol, steroids and secosteroids) have been considerably studied, because they may act as hormones or vitamins and have a four-ring structure. Interestingly, cholesteryl esters are more hydrophobic than cholesterol and tend to accumulate in the liver [18]. Prenol lipids are synthesized through the mevalonic acid pathway with the addition of terpenes and some derivatives (e.g., retinoic acid and isoprenoids) are important regulators of immune response [19]. Saccharolipids are fatty acids directly linked to a sugar backbone, and most are found in bacteria or plants [20]. Finally, polyketides are a large class of structurally and functionally diverse natural products with important bioactivities [21]. More information and references are usually needed to understand the significance of quantified lipids and may be documented via specific databases (http://lipidpedia.cmdm.tw) under construction. The physical and chemical properties of lipids are important to understand how they suit for their functions and also for avoiding putative limitations in the analysis. Here, we discuss some of the critical aspects involved in the goal to couple lipidomics and machine learning with the aim to propose pathogenic factors that may be used in the management of liver disease (Figure 3).

## 5. Sample Preparation and Systematic Error Removal

Lipids are usually amphipathic to build membranes, with one end positively charged and the other end formed by balanced ions, and this structure is prone to oxidation and hydrolysis. Immediate sample processing should be considered, but in clinical research, the procedure is generally not feasible. Serum and plasma samples must be flash frozen and stored at least at −80 °C, but for certain analysis, the effect of storage, when assessed experimentally, must be incorporated into the analysis of results as a covariate. Tissue samples or cells additionally require optimizing homogenization to ensure equal access of lipids to extraction solvents [22,23]. The extraction methods are designed to enrich the samples in lipids, removing nonlipid compounds, and need to efficiently reduce sample complexity and contamination, improving the signal-to-noise ratios and the identification rate. The efficiency of classical liquid–liquid extraction methods based on a mixture of chloroform–methanol and water is excellent for most purposes, but introducing acid or alkaline hydrolysis may maximize the extraction of certain lipids when time of extraction is critically considered [24,25]. Chloroform may also be replaced by methyl tert-butyl ether to facilitate handling and analysis of glycerophospholipids [26], and liquid–liquid extraction can be combined with solid-phase extraction to analyze gangliosides or ceramides [27]. To increase efficiency in positive electrospray ionization (ESI), derivatization is a successful strategy introducing isotopic labels or quaternary nitrogen atoms and methylating specific phosphate groups [28,29,30,31].

Multiple sample normalization is a crucial aspect in analytical strategies. Time-dependent drifts in instrumental sensitivity, as well as changes in pH and concentrations of solvents and temperature oscillations are common in studies that include many samples with a relatively long time of measurement and likely interruptions. Datasets are acquired over days or weeks, increasing the likelihood for multiple batches and systematic errors. Several software tools have been developed to aid in standardizing and automating the removal of systematic error that work in most lipidomics workflows [32,33,34]. Thus, the choice of internal standards and the use of pooled biological samples as quality controls measured in a ratio of approximately 10:1 are critical points in analytical strategies.

## 6. Addressing the Chemical Diversity of the Lipidome in a Biological System

No single analytical instrumentation is able to separate and identify all lipids. Multiple options are available, and ongoing research is constantly approaching its own advantages and limitations, but profiling the lipidome remains a challenging task. The improvement of analytical methods and development of software tools are both equally important to unravel the role of lipids into the fields of diagnostics and therapeutics. 

The number of analytical variations may be high, because there is a conflicting compromise between coverage, selectivity and throughput. The analysis of a crude lipid fraction by direct infusion ESI and nuclear magnetic resonance (NMR) generate large amounts of complex data with signal overcrowding and overlapping but do not require separation of lipids and provide fast analysis and easy implementation [35,36,37]. Reversed-phase high performance liquid chromatography (HPLC) is common, but recent technological step-changes have made mass spectrometry (MS) coupled to chromatographic separation techniques more reproducible, sensitive and easy to use. MS is the unrivaled technology in the field, and ultrahigh performance liquid chromatography (UHPLC) appears to be the golden analytical standard in lipidomics, although compounds that are volatile or can be made volatile are responsive to gas chromatography (GC) [38,39,40]. Hydrophilic interaction liquid chromatography (HILIC) is also frequently used, because this technique resolves lipids according to their polar head groups, and the solvents used are compatible with ESI [41,42]. Other options include supercritical fluid chromatography (SFC) or ultrahigh performance SFC (UHPSFC), which provides fast resolution time and high resolution [43]. High coverage pseudo-targeted lipidomics is a relatively novel emerging approach with potential value in lipidomics [44], and the incorporation of triple quadrupole-time of flight (q-TOF) mass spectrometers to high-resolution lipidomics platforms allow targeted lipidomics approaches with simple data processing steps [44,45]. Among other advantages, it is now possible to separate oxylipins from fatty acids with unambiguous interpretation [46,47]. More recent platforms also incorporate ion mobility spectrometry (IMS) and Ozone-induced dissociation (OzID) [48,49], which incorporate shape as an additional separation dimension through collision-cross sections (CCS) to identify the exact position of C=C [50,51,52]. An alternative method to determine double bond(s) position(s) is by coupling Paternò–Büchi reaction and MS [53,54]. The matrix-assisted laser desorption-time of flight (MALDI-TOF) may provide two-dimensional images of the distributions of lipids [55] and facilitates the identification of the double bond position and the sn position of the fatty acyls [56]. In each of these steps, standardized data quality check controls should be performed [57]. Thus, the Lipidomics Standard Initiative (https://lipidomics-standards-initiative.org Accessed on 3 March 2021) provides a simple framework to ensure that lipidomics analyses are as standard as possible and to improve their comparability. The addition of controls is also crucial in this regard. In addition, absolute quantification of data would be needed to transfer these applications to the clinical field. This overview of technical developments illustrates the rapidly changing field of lipid analysis. To date, some of the challenges imposed by the lipidome’s complexity are not entirely overcome. 

## 7. Extracting the Relevant Information

In lipidomics workflow, the management of a huge amount of data and their understanding is also a challenging task, which includes the acquisition of metadata. There is no laboratory information management system specifically tailored for lipidomics, and they remain partially adapted from those designed for metabolomics, but web-based methods are available, especially in an environment of R programming language [58]. Bioinformatics analysis of data starts once the samples are run in analytical instruments, because the interpretation of MS data requires retrieving all of its structural and functional content. Some bioinformatics packages may convert raw files into an accessible open format without incurring false positives, and lipid annotation may be performed using customizable lists based on experimental or computational methods [11,59,60,61]. Manual feeding into external lipidomics software is an alternative, especially when analytes are unknown or are found in low concentrations [62]. Relative quantification in case control studies is relatively simple, but absolute quantification may be mandatory when the aim is to compare data from different studies or laboratories [33]. 

Some workflow management systems have been designed as platforms with infrastructure to provide data analysis and algorithms that are appropriated for metabolomics [63,64,65]. Metaboanalyst 4.0 is widely used in metabolomics [66], and a companion R-package permits exploratory statistical and functional analysis, which are important to detect biomarkers or even predict NASH. There is room, however, to improve data integration and systems biology, because pathway mapping is still in its early stages in lipidomics, and the ultimate goal of lipidomics is to detect the role of lipids within metabolic pathways [67,68]. In this context, an additional barrier to interpret circulating lipidome in metabolic diseases is the constant and important communication among relevant organs.

## 8. Interorgan Communication in the Course of NAFLD

**Figure 4:**
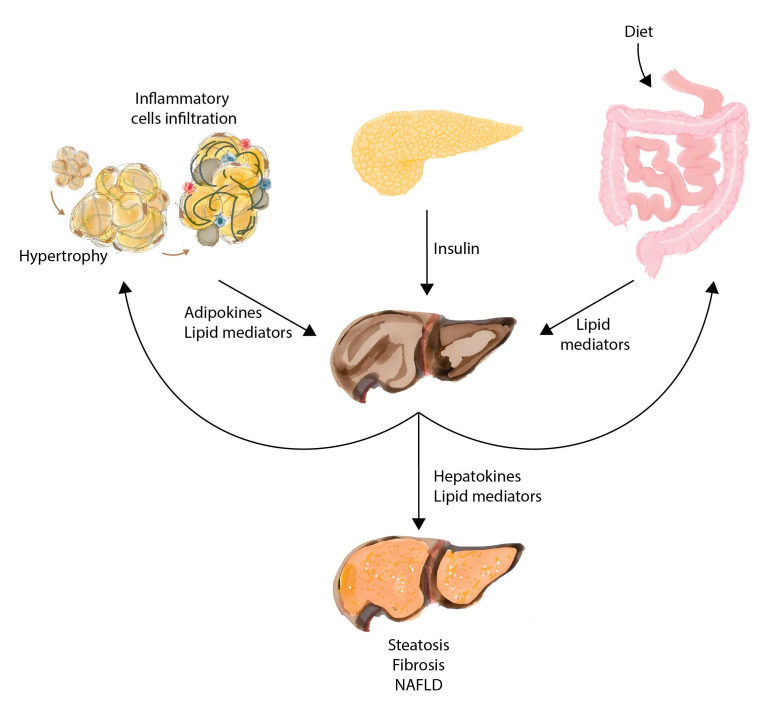
Lipidomics must consider the effect of interorgan crosstalk. Energy homeostasis is important in the health and disease of the liver and is highly dependent on metabolic signals derived from dietary intake or secreted from adipose tissue, gut, liver and all insulin-sensitive tissues.

NAFLD is a metabolic disease with associated comorbidities that include obesity (51% among NAFLD and 82% among NASH patients), diabetes, hypertension and dyslipemia [8]. Dysregulated glucose and lipid metabolism are the consequence of interrelated stimuli from at least the liver, the pancreas, the gut and the adipose tissue (Figure 4). Identified signals from the liver include lipids and hepatokines (e.g., fibroblast growth factor 21), which affect lipolysis and lipogenesis in target organs and contribute to insulin resistance [69,70]. The adipose tissue is now recognized as an endocrine organ, and signaling is disrupted in obesity through inflammation. The key actions of leptin, adiponectin and other adipokines may be critical in the onset of liver disease [71,72], and fatty acid-binding protein 4 specifically stimulates hepatic gluconeogenesis [73]. “Organokines” affect each other and communicate through endocrine, paracrine and autocrine pathways. The actual mechanisms of action and metabolic consequences of lipid signals, lipokines, delivered by the interconnected organs, are unknown, but their effects on systemic metabolism support the hypothesis that lipid factors may influence, as cause or effect, the progression of chronic liver diseases [74,75]. These findings also support efforts on the field of lipidomics and NAFLD.

Lipotoxic or glucolipotoxic liver injuries are key events in NAFLD pathogenesis and progression [76,77]. Clinically, NAFL has an apparently indolent course, and prognosis is more favorable than NASH, but NAFL is more progressive than previously thought, and it is no longer considered a benign condition [78]. The data associated with long-term outcomes are scarce, because accurate diagnosis needs histological assessment. However, the incidence of hepatocellular carcinoma in NAFLD patients appears to be 0.44 per 1000 person-years and 5.3 per 1000 person-years in patients with NASH [8], which is extremely important in a disease affecting millions worldwide. The presence and stage of fibrosis are the most important predictors of complications in NAFLD [79,80], and age, obesity and inflammation on initial liver biopsy are independent factors associated with progression to advanced fibrosis [81]. Efforts to find noninvasive procedures to diagnose NAFLD or to predict NASH cannot be overstated in a disease without accepted cure. Bariatric surgery has demonstrated its potential to reverse NASH, diabetes and other associated comorbidities [82,83,84,85,86]. The actual mechanisms are unknown, and they are both dependent and independent of weight loss, but available data are extremely suggestive. The challenge remains in surgical procedures, and a lipidomics approach may add knowledge of metabolic regulation and potential therapeutic targets.

## 9. Can Lipidomics Provide Insights into the Pathogenesis of NAFLD?

Most data have been obtained in animal models, but results in mice have been traditionally difficult to interpret, and their contribution to understanding the disease remains debatable [87]. For now, the pathogenesis of NAFLD in humans remains perplexing and open to new perspectives. Human lipidomics in NAFLD has been reviewed recently [13]. The contribution to circulating lipidome from changes in hepatic lipid composition or from the diversity in lipid composition of visceral and subcutaneous adipose tissue have not been resolved in liver disease. Lipid profiles in portal circulation, a drain for lipids from visceral organs to the liver, might support gut–liver interactions through the hepatic exposure to microbial endotoxins [88,89].

The first human studies in liver biopsies identified in NASH patients, compared to NAFL patients, differences in fatty acid and phospholipid composition, which are important to ensure membrane integrity, especially in mitochondria [12,90,91,92,93]. However, the assumption that, under fasting conditions plasma lipids reflect the lipids exported from the liver, remains incompletely understood [94,95,96,97,98]. The diversity of phenotypes in NAFLD is considerable, and not all individuals with NAFLD are insulin resistant, become diabetic or progress to NASH. Thus, matching cohorts remains a major challenge. Results among studies are also difficult to compare, because there is a significant overlap between plasma and liver lipidomes [99,100], the crucial role of mitochondrial dysfunction in NAFLD pathogenesis is difficult to assess [101,102] and the presence of covariates in NAFLD are not always adjusted [103]. A monozygotic twin study indicated that circulating lipidome was independent of genetic effects, but obesity and hypertension, for which was not adjusted, were associated with changes in phospholipid metabolism and saturated fatty acids [104,105,106]. Lipidomics might be a tool to propose strategies aimed to identify those at greatest risk of developing NASH and to understand the pathways and networks involving lipids and their metabolism [32,107,108,109,110].

Mitochondrial lipids and the communication with lipid biosynthesis in endoplasmic reticulum may cause liver disease via defective mitochondrial function, which is highly dependent on a regulated supply of phospholipids and proteins [86,100,101,111]. In the clinical context of oversupply of nutrients and obesity-associated NAFLD, lipidomics demonstrates the interaction among hepatic lipid and glucose metabolism, oxidative stress and inflammation via bioactive lipid mediators [112,113,114,115,116]. Several results prompt the study of polyunsaturated fatty acids of phospholipids and their conversion to bioactive lipid mediators through the cyclooxygenase and lipoxygenase pathways. In obesity, remodeling of glycerophospholipids in membranes may represent an adaptation of adipocytes to facilitate the store of increased fat content [113]. Mice are sensitized to liver injury through oxysterols (cholesterol-derived products) and lipid mediators derived from arachidonic acid (e.g., eicosanoids), resulting in proinflammatory and profibrogenic effects [117,118,119]. The pathogenic role of these lipids in the course of NASH and fibrosis is currently under investigation [120]. Conversely, oxylipins (proinflammatory products of PUFA metabolism) are increased in plasma of NAFLD patients but are not responsive to gut-derived immunogens [121]. Recently, specialized pro-resolving mediators have been involved in the resolution of hepatic inflammation and fibrosis [122].

Patterns are simply impossible to identify in the absence of computers and software with the ability to build the relationships among hundreds of lipid species and to resolve biochemical mechanisms underpinning altered lipidomes and their metabolic implications [109,123,124,125,126]. Data in patients with NAFLD involve a complex constellation of changes that occur dynamically and vary from patient to patient. In the future, the ability to collect and analyze large datasets promises to transform medicine, with implications for disease diagnosis and treatment [63,127]. Below, we briefly discuss methods coupling the field of metabolomics and lipidomics with machine learning and the potential to provide noninvasive alternatives to manage chronic liver diseases.

## 10. Machine Intelligence and Learning Approaches

**Figure 5:**
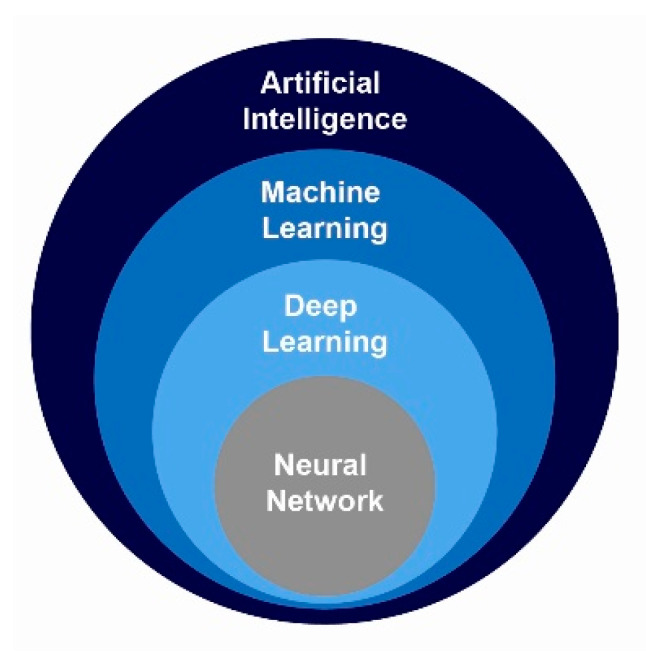
Deep learning is a type of machine learning. Expert systems can exceed human-level achievements in the diagnosis of a disease.

The use of pencils and a calculator are no longer valid strategies to manage results. Artificial intelligence is an absolute requirement to unlock current biomedical datasets, and the concept refers to a broad class of systems that enable machines to mimic or exceed human capabilities. Machine learning (ML) is the most common way to achieve artificial intelligence using data to predict outcomes, and deep learning (DL) is a special type of ML that may discover relevant features from labeled data using a “neural network,” a name inspired by a mathematical object called artificial neuron. Their relationship is shown in Figure 5. In the age of “big” data, ML is a discipline in computer science, wherein machines (computers) can learn patterns from data, and the learned model(s) can be used to predict outputs [128,129,130]. In science and biomedicine, ML can find predictive patterns to understand complex biological systems and is currently used in lipidomics to process the amount of data generated by modern mass spectrometry [131]. In the context of metabolic studies, we can create a predictive model that predicts a given metabolite according to the peak detection and may improve diagnostic accuracy and treatment variability to make progress under a clinical approach [132,133,134,135]. To establish predictions, a common practice in ML is to evaluate an algorithm by splitting a dataset into the training and the testing set with techniques that fall into a few categories but require high quality data and previous selection of the importance of features [136]. There are many types of learning, but those more popular in biomedicine are broadly divided into supervised, in which the outcome of the training data is already known, and unsupervised techniques that operate the data without knowing the outputs or target variables and without correction. More recently, a semisupervised learning approach is devised to combine both techniques: a part of the introduced dataset has an unknown outcome, and the other one is already labeled with its corresponding category. Unlike other types of learning, when using reinforcement learning algorithms, the system is not trained with the sample. Rather, the system learns through trial-and-error, interacting with an environment and learning from its experiences.

**Figure 6:**
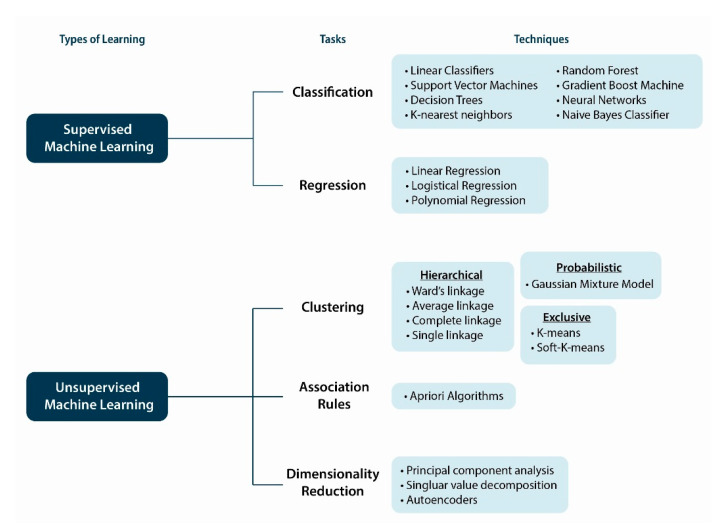
Tasks and techniques used in machine learning. There are many types of learning, but supervised and unsupervised machine learning types are already available and relatively easy to use in biomedicine.

Task and techniques used in supervised and unsupervised learning are summarized in Figure 6. In supervised learning, ML applications generate trained models that may be predictive. The outputs may be classificatory, to predict discreet categories (e.g., healthy versus NAFL, NAFL versus NASH), or, in linear models, are similar to regression, in which outputs may predict the value of other continuous variables. Methods are also used to infer statistical conclusions. Partial least squares (PLS) regression and its variants, PLS discriminant analysis (PLS-DA), orthogonal PLSDA and sparse PLSDA have been used to explain variation in metabolomics [137]. Other ML techniques include neural networks, naive Bayes, support vector machines, random forests, kernel machines, Bayesian networks or fuzzy logic [138,139]. These learning models avoid additional time in classification and can make predictions, but overfitting data is a common risk. Unsupervised approaches are suited for clustering, association and visualization of high-dimensional input data allowing exploratory analysis of similarities and differences between groups. The labels on the input data are unknown and learn only from patterns (clusters) in the features of the input data. A predictive model is not produced but may determine where potential new data fit with respect to the original data. Commonly used methods include clustering (exclusive, overlapping, hierarchical, and probabilistic) and dimensionality reduction (principal component analysis (PCA), singular value decomposition, autoencoders) algorithms. Unlabeled data are categorized to identify patterns and can be useful for image detection and diagnostic purposes. Challenges to resolve in these methods include the risk of low accuracy and time-consuming validation. The discovered and validated clusters can be used as input features to supervised methods.

Ensemble models tend to be the most robust, although the simple is often better, but there is no ML method that will optimally solve all ML tasks in network biology. The interpretability of PLS model is high and represents an effective hybrid prediction–inference algorithm for high dimensional data, which depends on the field of study. Artificial neural networks are ML tools based on interconnecting hidden layers, computational structures inspired by neurons in the brain, and in their simplest forms are similar to PLS but can model nonlinear models. Deep neuronal networks, or DL, can predict relationships from diverse datasets and can accomplish supervised, semisupervised and unsupervised tasks, improving the interpretability of data analysis [140,141,142,143]. DL techniques transform the data by iteratively tuning their internal parameters and may enable the extraction of the most predictive features from complex datasets. A selection of open-source tools for ML based on DL architectures may be found elsewhere [131].

Rather than simply identifying potential biomarkers, ML algorithms may help define the underlying mechanisms exploring the dysregulation of networks leading to disease state [144]. The mummichog framework can predict the functional activity of a metabolic pathway and Lilikoi, an R-package, can personalize pathway-based classification modeling using metabolomics data [145,146]. It is also possible to integrate multi-omics data, which provides more useful understanding of biology [147]. Conventional ML techniques may require specific analytics platforms that have enabled successful integration to predict relevant information in biomedicine and clinical management [148,149,150]. We next highlight a recent proof of concept study that paves the way to coupling lipidomics and ML to predict models for diagnosing NAFLD.

## 11. Predicting the Risk of NASH with Lipidomics and Machine Learning

There are some noninvasive scores combining clinical variables and laboratory measurements that were derived from patients with advanced liver disease and describe associations with liver fat accumulation, but the predictive power is poor in the management of NAFLD, obesity and diabetes [151,152]. Liver ultrasound is widely used for the diagnosis of significant liver fat accumulation, which is also noninvasive but with major drawbacks in obese patients [153]. The reliability and limitations of other imaging techniques remain to be established in clinical practice for the management of NAFLD [154]. None of these techniques and clinical variables can differentiate NAFL from NASH. Thus, NASH remains undiagnosed if a liver biopsy is not performed.

In the context of noninvasive diagnosis, lipidomics may integrate metabolic pathways and provide a unique perspective of liver fat accumulation. Data obtained from the coupling of lipidomics and ML has been tested recently for the first time under a hypothesis-driven research [155,156]. This was a pilot study in a limited number of patients that needs prospective confirmation, but they could predict the presence of liver fibrosis with high accuracy. The authors analyzed 365 lipids, 61 glycans and 23 fatty acids in healthy subjects and patients with NAFLD or NASH and found that One-vs.-Rest support vector machine models with recursive feature elimination identified 29 lipids or combinations between lipids, glycans and hormones could differentiate with very high accuracy (up to 90%) between the three conditions. In an exploratory analysis, a model consisting of 10 lipid species could robustly discriminate between the presence of liver fibrosis or not (98% accuracy). These data prove that a lipidomic approach is potentially useful to predict NAFLD outcome without liver biopsy. More important, ML tools were simple and easy to perform in freely available platforms in python and in R operating systems. Other ML and DL platforms with open-source software are also available (Table 1).

Despite the limitations, this proof-of-concept study illustrates the challenges researchers in the field of precision medicine face, in this case, accurate, noninvasive diagnosis of NASH via multi-omics data integration. Indeed, data integration algorithms are available to integrate anthropometric and clinical chemistry data to multi-omics data but may be improved in future developments [157,158,159]. To increase sample size, large clinical assays have prohibitive costs and are time-consuming. As an alternative, there are several repositories open to collaboration, and biological samples may be distributed upon request. Under this approach, ML methods are also useful to integrate known and unknown factors of heterogeneity using techniques to both minimize error and maximize efficiency. We envision a future in which the goal of clarifying mechanisms underlying NASH may be fulfilled using multi-omics technology to facilitate therapeutic solutions, and opportunities are likely located at the intersection of network biology and machine learning.

## 12. Conclusions

Compared to another omics fields, lipidomics remains in an early stage. Its use in the field of hepatology requires substantial improvements in knowledge, technical analysis and software developments. The progressive nature of NAFLD is apparently established and associated with high morbidity and mortality, but there are not specific treatments. The outcomes of NASH may be cirrhosis and hepatocellular carcinoma, two conditions with poor prognosis that may lead to liver transplantation. Unfortunately, NASH remains undiagnosed if a liver biopsy is not performed. Lipidomics integrate metabolic pathways and provide a unique perspective of NAFLD. Mass spectrometry is the unrivaled technology in the field, and every technical aspect in lipidomics analytic measurement requires machine intelligence. Studies in NAFLD lipidomics result in a tremendous amount of data, hampering the identification of useful patterns with the ability to resolve biochemical mechanisms underpinning altered lipidomes, to facilitate the comprehensive analysis of hundreds of lipid species and to understand the metabolic implications. Machine learning has the potential to unlock large biomedical datasets. Coupling lipidomics and machine learning methods, and, possibly, network biology, may provide predictive models searching noninvasive diagnostic alternatives and novel therapeutics.

## Figures and Tables

**Table 1:** Overview of the main open-source machine and deep learning frameworks.

Frameworks	Programming Languages	Features
Apache Spark	Java, R, Python, Scala	Structured data processing for machine learning and graph processing.
Caffe	C++, Python	Supports different deep learning architectures like CNN or RNN.
Chainer	Python	Provides a flexible, intuitive and high performance of deep learning models, such as RNN and autoencoders.
Deeplearning4j	Java	Works with different data types, such as images, CSV, plain text, audio and video to build a full range of deep neural network.
h2o.ai	Java, R, Python, Scala	Provides fast and scalable machine learning and predictive analysis platform.
Keras	Python	It is a deep learning API that works with machine learning platform TensorFlow.
Neon	Python	Artificial intelligence platform that works with images and videos.
Pytorch	C++, Python	It is a Python library for deep learning that provides fast and flexible framework to build dynamic neural network.
Scikit-learn	C, C++, Python, Cython	It is library for machine learning and statistical modeling that supports supervised and unsupervised learning.
TensorFlow	C++, Python	Machine learning platform that builds API for implementing machine learning, deep learning and science computing models.
Theano	Python	It is a Python library that provide train deep neural networks algorithms.

## Data Availability

Not applicable.
